# Headache in Calcified Neurocysticercosis: Emerging Insights into an Inflammation-Driven Phenotype

**DOI:** 10.4269/ajtmh.26-0374

**Published:** 2026-10-20

**Authors:** Oscar H. Del Brutto

**Affiliations:** Research Center, Universidad Espíritu Santo–Ecuador, Samborondón, Ecuador

## Abstract

Calcified neurocysticercosis (NCC), traditionally considered an inert marker of past infection, is increasingly recognized as a clinically meaningful condition associated with migraine-like headaches. Evidence from population-based cohorts, clinical series, and imaging studies consistently shows that individuals with calcified NCC have a higher prevalence and severity of headache than those without lesions. Prospective neuroimaging demonstrates that intermittent perilesional inflammation, including edema and blood–brain barrier disruption, can activate migraine-generating pathways, with calcitonin gene-related peptide-mediated trigeminovascular sensitization emerging as a key mechanism. These findings challenge the assumption that calcified lesions are clinically irrelevant and instead, support the concept of a distinct inflammation-driven headache phenotype. Recognizing this entity has important implications for diagnosis, counseling, and therapeutic decision-making, particularly in endemic regions where calcifications are common and often dismissed as incidental. Calcified NCC also provides a unique human model for understanding how focal neuroinflammation shapes headache susceptibility.

Neurocysticercosis (NCC), which is caused by the encysted larval stage of *Taenia solium*, is a leading cause of acquired epilepsy in endemic regions of Latin America, sub-Saharan Africa, Southeast Asia, and the Indian subcontinent.^1^^,^^2^ Headache is also a frequent neurological symptom in NCC,^3^ but for decades, it was considered a consequence only of severe or space-occupying forms of the disease. Classical descriptions emphasized that headaches occurred primarily in patients with subarachnoid cyst clusters, intraventricular cysts, giant parenchymal cysticerci, or cysticercotic encephalitis, where increased intracranial pressure, hydrocephalus, or meningeal inflammation provided an obvious pathophysiological substrate (Figure 1).^4^ This traditional framework relegated headache to a secondary manifestation of advanced disease.

**Figure 1.**
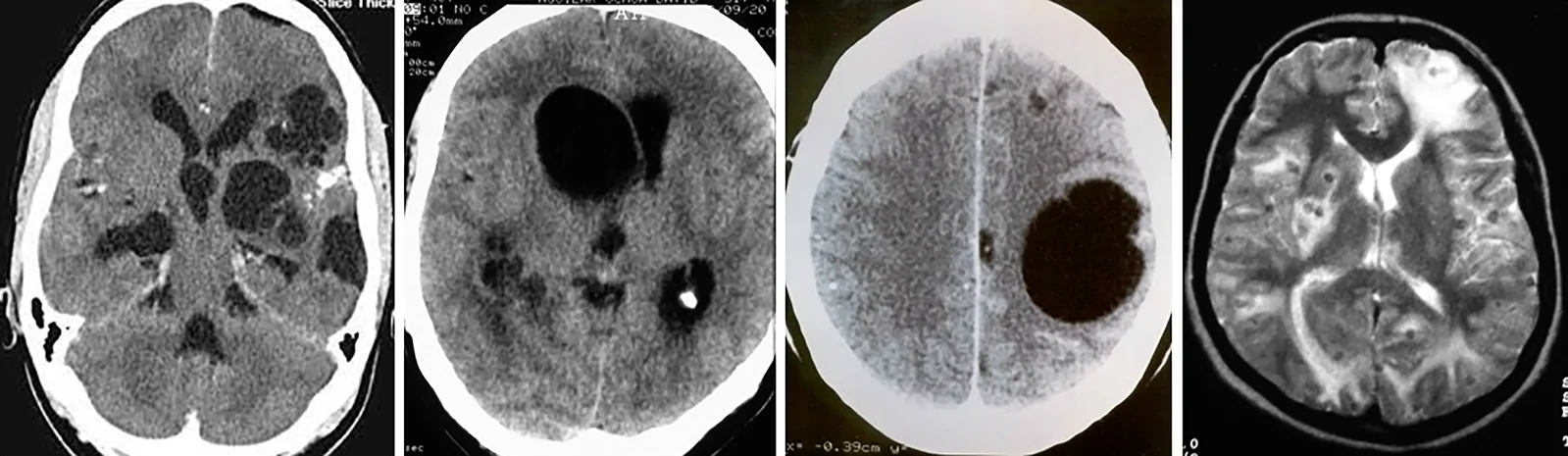
Severe forms of neurocysticercosis traditionally associated with headaches. From left to right, the images show clumps of subarachnoid cysts, large intraventricular cysts, giant parenchymal brain cysticercus, and cysticercotic encephalitis.

Over the past years, however, a markedly different scenario has emerged. A growing body of epidemiological, clinical, and mechanistic evidence indicates that calcified NCC—the end stage of infection—is not clinically silent but is significantly associated with primary headache disorders, particularly migraine. This shift has profound implications because calcified NCC is far more prevalent than active disease and has long been regarded as an inert and clinically irrelevant finding.^5^ The accumulated data now challenge this assumption and instead, point toward a dynamic, intermittently inflammatory biological process underlying the association between calcified NCC and headache.

The first major indication that calcified NCC might be linked to headaches in the absence of increased intracranial pressure came from community-based research in a small Andean village of Ecuador.^6^ That study identified a markedly higher prevalence of calcified NCC among individuals with migraine; one third of migraine sufferers had computed tomography-detected calcifications compared with ∼13% of headache-free controls (odds ratio [OR]: 3.4; 95% CI: 1.6–7.5). These findings were striking not only because of the magnitude of the association but also, because they emerged from a true population-based sample, minimizing referral and diagnostic bias and providing early evidence that even “inactive” lesions may have clinical relevance.

Subsequent work in a large clinical cohort further strengthened the epidemiological signal. In a study of more than 1,000 patients with headache evaluated at a single center in Ecuador, 4.7% had calcified NCC compared with 1.8% of neurological controls.^7^ The OR of 2.7 (95% CI: 1.6–4.3) remained significant across headache subtypes, indicating that the association is not restricted to a single clinical phenotype. Importantly, the association persisted even when compared with control groups that themselves had neurological disease, suggesting that the relationship is unlikely to be explained by differential imaging rates or referral patterns.

A case–control study nested within a well-defined population-based cohort in rural coastal Ecuador showed that individuals with calcified NCC consistently reported higher rates of lifetime headache (OR: 4.2; 95% CI: 1.8–9.8), current headache (OR: 4.2; 95% CI: 1.9–9.2), and intense headache (OR: 9.5; 95% CI: 2.9–31.2) compared with matched controls.^8^ These findings, which were derived from a population in which NCC transmission has been spontaneously arrested,^9^ provide compelling evidence that calcified NCC is a clinically meaningful and independent risk factor for headache. This unique epidemiological context—free from the confounding effects of active disease or reinfection—offers a rare opportunity to isolate the clinical consequences of calcified cysticerci.

Other hospital-based studies have also supported this association. A case–control study from India showed that headaches were more frequent and more severe in migraineurs with calcified NCC than in those without.^10^ Interestingly, the response to prophylactic treatment with amitriptyline was better among patients with calcified NCC, possibly related to the anti-inflammatory properties of this drug. In another study of 160 individuals with epilepsy attending a neurologic clinic in California (39% with NCC), headaches were significantly more common among those with NCC.^11^ In contrast, a separate Indian case–control study reported lower migraine frequency in patients with calcified NCC than in those without.^12^ However, this outlier has been attributed to substantial selection and recruitment biases,^10^ and its methodology limits comparability. Overall, the hospital-based literature aligns with population-based findings, reinforcing the notion that calcified NCC is not a benign radiological bystander but a lesion with potential clinical consequences.

A recent systematic review and meta-analysis synthesized data on NCC and headache from population-based cohorts, clinical series, and case reports.^13^ In that study, all forms of NCC—not only calcifications—were pooled, which limits the precision with which the specific contribution of calcified lesions can be isolated. Even so, the review provides important corroborative evidence. Across studies, individuals with primary headache disorders were nearly three times more likely to have calcified NCC (OR: 2.8; 95% CI: 1.8–4.5), and individuals with calcified NCC were similarly more likely to report primary headaches (OR: 2.8; 95% CI: 1.9–14.2). Although the meta-analysis did not identify consistent differences in headache severity, the epidemiological signal remained stable across heterogeneous settings.

Although most patients with NCC-related headache have calcified lesions, acute headache can also be the sole manifestation of another relatively benign form of NCC, the so-called solitary cysticercus granuloma (SCG). In a prospective series from India, 2.5% of patients with SCG presented exclusively with severe episodic headache, often accompanied by vomiting but without seizures.^14^ These episodes occurred during the degenerating phase of the cyst when perilesional edema and inflammatory enhancement were most prominent. Once the granuloma resolved, the headaches disappeared. These cases demonstrate that even a single degenerating cyst can provoke intense, migraine-like episodes, illustrating how transient inflammatory activity can directly trigger headache.

The question then is why calcified cysticerci—long regarded as biologically inert—would provoke headaches. Several mechanistic pathways have been proposed, and recent imaging studies suggest that calcified lesions may retain the capacity to trigger intermittent inflammatory activity capable of activating headache-generating pathways. The most influential concept is that calcified cysticerci may undergo intermittent remodeling, during which residual parasitic antigens become exposed to the host immune system.^15^^,^^16^ This antigenic release can trigger focal inflammatory responses, leading to blood–brain barrier disruption, vasogenic edema, and activation of nociceptive pathways. The resulting inflammatory cascade may also generate oxidative stress, further sensitizing adjacent neural tissue.

One of the key mediators implicated in this process is calcitonin gene-related peptide (CGRP), a neuropeptide central to migraine biology.^17^ Inflammatory stimuli around calcified lesions may upregulate CGRP expression, lowering the threshold for trigeminovascular activation and facilitating migraine-like attacks.^18^ Notably, CGRP may play a dual role; although it can promote migraine activation, it may also help stabilize a disrupted blood–brain barrier, potentially reducing the risk of breakthrough seizures associated with perilesional inflammation (Figure 2).

**Figure 2.**
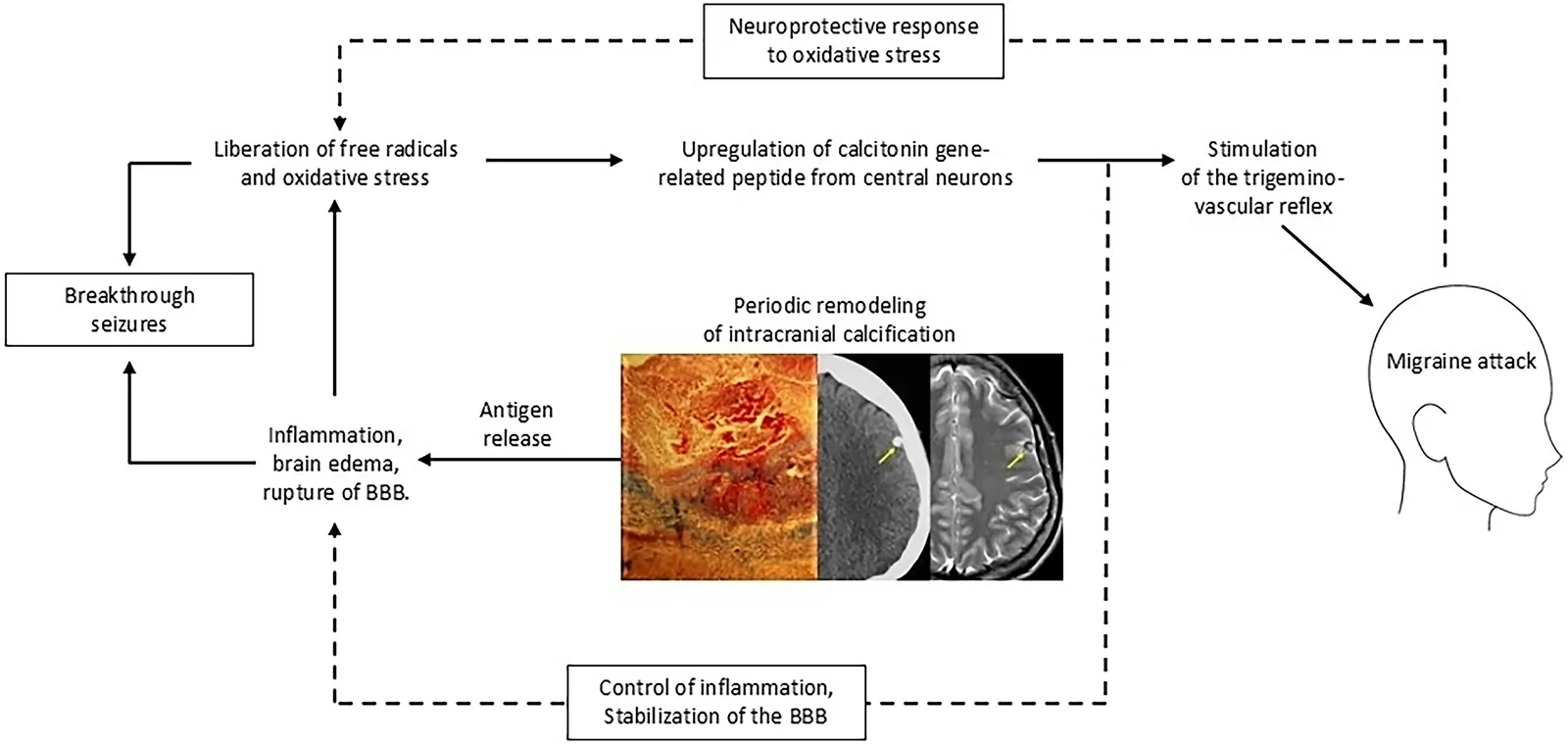
Proposed sequence of events leading to migraine-like attacks in patients with calcified neurocysticercosis. BBB = blood-brain barrier. Adapted from Del Brutto et al.^18^ (published under the Creative Commons CC BY-NC license).

The role of perilesional edema as a direct trigger of migraine-like attacks has been underscored by prospective imaging studies. In a cohort of 80 patients with migraine and calcified NCC, 6 individuals (7.5%) exhibited perilesional edema.^19^ These patients experienced an average of ∼23 headache days per month compared with ∼8 days in those without edema, and they reported greater disability. Although the edema resolved, the affected individuals continued to experience more severe headaches, suggesting lasting sensitization of headache pathways. This study provides strong evidence that perilesional inflammation is not an incidental radiological finding but a key biological driver of headache in calcified NCC.

Despite the growing clarity of the association, several key questions remain unresolved. It is unknown whether headaches begin only after calcification or whether individuals may be predisposed earlier in the disease course. The temporal relationship between perilesional edema and headache episodes also remains uncertain as prospective neuroimaging during attacks is rarely feasible. The potential impact of CGRP-targeted therapies, which are now widely used for migraine, has not been evaluated in NCC, although there is currently no evidence suggesting harm. It is likewise unclear whether the number, size, or anatomical distribution of calcifications influences headache risk or whether host-specific factors modulate susceptibility.

According to “The International Classification of Headache Disorders, 3rd edition (beta version),” headaches occurring in the context of a persistent structural brain lesion, such as calcified NCC, are classified as secondary headaches, even when their phenotype resembles primary migraine.^20^ The requirement for a temporal association and resolution within 3 months applies only to acute symptomatic headaches, not to those arising from chronic, nonresolving intracranial lesions. Thus, in patients with calcified NCC, the attacks are best described as migraine-like episodes attributed to a chronic intracranial lesion. This distinction underscores that phenotypic similarity does not imply identical pathophysiology, and it raises the question of whether current classification frameworks adequately capture inflammation-driven headache phenotypes.

Taken together, these findings support a coherent and biologically plausible model in which calcified NCC is not a passive marker of past infection but an active contributor to headache susceptibility. The evidence suggests that intermittent inflammatory activity around calcified lesions can activate migraine-generating pathways, particularly in individuals with a predisposing neurobiological profile. This framework reconciles the paradox of “inactive” lesions producing recurrent symptoms and aligns with broader concepts of neuroinflammation in primary headache disorders. It also underscores that calcified NCC represents a distinct, inflammation-driven headache phenotype, one that may not be fully captured by current classification systems.

In summary, calcified NCC is a clinically meaningful condition capable of provoking migraine-like headaches through intermittent, inflammation-driven mechanisms. Recognizing this entity has important implications for diagnosis, counseling, and therapeutic decision-making, particularly in endemic regions where calcifications are highly prevalent and often dismissed as incidental. Future research should clarify the temporal dynamics of perilesional inflammation, identify biomarkers of susceptibility, and determine whether targeted anti-inflammatory therapies may benefit selected patients. More broadly, calcified NCC offers a unique human model for understanding how focal neuroinflammation shapes headache phenotypes.
